# Male contraceptive efficacy of poly herbal formulation, contracept-TM, composed of aqueous extracts of *Terminalia chebula* fruit and *Musa balbisiana* seed in rat

**DOI:** 10.1080/13880209.2017.1357734

**Published:** 2017-08-24

**Authors:** Abhinandan Ghosh, Bhabani Prasad Pakhira, Adrija Tripathy, Debidas Ghosh

**Affiliations:** Nutrigenomics & Molecular Medicine Laboratory, Department of Bio-Medical Laboratory Science and Management, Vidyasagar University, Midnapore, India

**Keywords:** Androgenesis, antitesticular activity, cyproterone acetate, oxidative stress, spermatogenesis, sperm motility, sperm viability, steroidogenesis

## Abstract

**Context:***Terminalia chebula* Retz (Combretaceae) and *Musa balbisiana* Colla (Musaceae) have a traditional reputation as a male contraceptive.

**Objective:** To determine the hypo-testicular activity of aqueous extracts of *Terminalia chebula* (fruit) and *Musa balbisiana* (seed) separately, and in composite manner at the ratio of 1:1 named as ‘Contracept-TM’ compared to cyproterone acetate (CPA), for developing a polyherbal contraceptive.

**Materials and methods:** The separate extract of above said plants or ‘Contracept-TM’ at the dose of 40 mg/100 g body weight of rat/day or CPA at 2 mg/100 g body weight of rat/day was administered for 28 days. Spermiological, androgenic and oxidative stress sensors, LD_50_ and ED_50_/100 g body weight values were measured.

**Results:** Treatment of individual, ‘Contracept-TM’ or CPA resulted significant decrease in the count of spermatogonia A (36.36–49.09%), pre-leptotene spermatocyte (19.11–55.30%), mid-pachytene spermatocyte (28.65–47.28%) and step 7 spermatid (29.65–51.59%). Activities of testicular Δ^5^, 3β (21.25–48.02%),17β-hydroxysteroid dehydrogenases (29.75–55.08%), catalase (19.06–43.29%) and peroxidase (30.76–62.82%), levels of testosterone (28.15–63.44%), testicular cholesterol (19.61–49.33%), conjugated diene (29.69–84.99%) and thiobarbituric acid reactive substances (41.25–86.73%) were elevated compare to the control. The ED_50_ and LD_50_ values were 40 mg and 5.8 g (*T. chebula*), 48 mg and 6.3 g (*M. bulbisiana*), 40 mg and 6.0 g (‘Contracept-TM’), respectively.

**Discussion and conclusion:** The said spermiological and androgenic sensors’ levels were decreased significantly by ‘Contracept-TM’ than its constitutional individual plant extract and it may be comparable to standard anti-testicular drug like CPA. So, it may be concluded that above polyherbal formulation is potent for inducing hypo-testicular activity.

## Introduction

The world population explosion has pointed out the need for new, effective and safe contraceptive agents for maximum protection against fertilization. Side effects of existing synthetic contraceptives on the human body are increasingly aggressive and unpredictable at prolonged use. Women have a wide range of contraceptive choices ranging from daily oral medication to intrauterine devices implanted every 5 years (Roy et al. 2009). Research and family planning organizations have focused upon female methods of contraception for a long time because women bear a disproportionate portion of health and economic consequences of childbearing and rearing. The consequence of this long negligence for producing acceptable and reliable male contraceptives in developing countries results lower participation of males in family planning. Neglecting males in matters of family planning is a losing strategy with adverse consequences for both men and women (Ringheim 1993). The two most common male contraceptive methods are surgical (i.e., vasectomy) and physical barrier for sperm delivery (i.e., condom). The disadvantages of these methods are that vasectomy is not readily or easily reversible whereas condoms have a high typical failure rate.

Therefore, it is now time to think of an alternative in the field of male contraception. Accordingly, efforts are being made to explore the efficacy of plant products as a potential male contraception. There are many references covering plants with antifertility properties in the literature (Chinoy et al. 1995; Zhen et al. 1995; Ghosh et al. 2002; Jana et al. 2003). For instance, an *in vitro* study on human sperm has revealed that a composite extract of *Acyranthes aspera* Linn (Amaranthaceae) and *Stephania hernandifolia* Willd (Menispermace) acts as a spermicidal agent (Paul et al. 2006, 2010). *Terminalia chebula* Retz (Combretaceae) has a long folk medicine reputation for induction of male infertility. Extracts of *T. chebula* are rich in flavonoids and have been found to possess antispermatogenic activity in both humans and rats (*in vivo* and *in vitro*) (Srivastav et al. 2010). *Musa balbisiana* Colla (Musaceae) has traditional and folk medicine reputations as a male contraceptive in India (Das et al. 2014).

In herbal treatment, a combination of different plant extracts is used for the correction of a particular health disorder. Such combinations exhibit better results than a single plant extract through herb–herb interaction (Wu et al. 1998; Chatterjee et al. 2009). Our laboratory previously reported antifertility effect of hydro-ethanol extract of *Terminalia chebula* in male rat (Ghosh et al. 2015). The present study investigates the most effective antifertility property of aqueous extract of fruits of *T. chebula* and seeds of *M. balbisiana* in separate and in composite way (‘Contracept-TM’) (1:1) of said parts of two plants in respect to standard antifertility drug, cyproterone acetate (CPA).

## Materials and methods

### Preparation of plant extract

*Terminalia chebula* fruits and *M. balbisiana* seeds were collected (June–July, 2015) from the local market (Goaltore, Paschim Medinipore) of Midnapore district by research scholar (A. Ghosh). Both the plant species were identified and authenticated by Dr. Ram Kumar Bhakat, Associate Professor, Department of Botany of our University. The voucher specimens were deposited in the Herbarium of the Botany Department of Vidyasagar University. *T. chebula* fruits and *M. balbisiana* seeds were dried, powdered and extracted separately in distilled water at 37 °C for 24 h. Individual extract was filtered and the resultant filtrate was dried. ‘Contracept-TM’ was prepared exclusively by mixing the extracts of *T. chebula* and *M. balbisiana* in a ratio of 1:1.

### Experimental animals

Thirty Wistar strain male albino rats, three month of age and weighing about 120 ± 10 g were used for the present study. Animals were housed in cages at an ambient temperature of 25 ± 2 °C under 12 h light-dark cycles and were kept for 15 days for acclimation prior to experimentation. They were provided with standard rat chow diet and water *ad libitum*. The Institutional Ethics Committee (IEC) approved the study and all the instructions given by our IEC [IEC/4-C-6/35] were followed throughout the experimentation. Another 20 male adults rats were used for LD_50_ and ED_50_ studies.

### Experimental design

Ten adult male rats were used for LD_50_ study and another ten adult male rats were used for ED_50_ study. For antitesticular activity of the said extracts, 30 Wistar strain albino rats after 15 days of acclimation were used and the body weight of each rat was recorded. Thereafter, rats were divided into five groups, six animals in each group. The daily dose of the extract, and CPA were prepared by suspending in water and administered to each animal through oral route by gavage in the morning (at 8.00 AM). The duration of the experiment was 28 days. The treatment schedule of each group was as follows:

***Group I****(Vehicle treated control):* Rats of this group received 0.5 mL of distilled water/100 g body weight once a day.

***Group II****(T. chebula treated group):* Rats were treated with the aqueous extract of *T. chebula* at the dose of 40 mg/0.5 mL water/100 g body weight once a day.

***Group III****(M. balbisiana treated group):* Animals of this group were treated with the aqueous extract of *M. balbisiana* at the dose of 40 mg/0.5 mL water/100 g body weight once a day.

***Group IV****(Composite (1:1) T. chebula and M. balbisiana or ‘Contracept-TM’ treated group):* Rats were treated with composite aqueous extract of *T. chebula and M. balbisiana* at the ratio of (1:1) i.e., ‘Contracept-TM’ and at the dose of 40 mg/0.5 mL water/100 g body weight once a day.

***Group V****(Cyproterone acetate treated group):* Animals of this group received 2 mg of CPA/0.5 mL water/100 g body weight once a day.

After completion of the experimental schedule, all the animals were subjected to light ether anaesthesia after recording the body weight. Blood was collected from dorsal aorta of each animal just after anaesthesia and serum was separated by centrifugation at 3000 *g* for 5 min and stored at −20 °C for testosterone assay. Then animals were decapitated one by one using sharp knife. Reproductive organs i.e., testes, epididymis and seminal vesicles were dissected out. Fat and other connective tissues were removed from the surface of the organs and wet weights of these organs were recorded. Left testis of each rat was kept at −20 °C for enzymatic study and the right testis of each animal was placed in Bouin’s fluid for histological study.

### Epididymal sperm count, sperm motility and sperm viability assessment

Spermatozoa were collected from an equal length of the cauda of the excised epididymis of each rat of all the groups by flushing it with the same volume (2 mL) of a suspension medium. The count for spermatozoa in a 100 µL suspension under microscope was performed with hemocytometer following the standard method and expressed as the number of spermatozoa per mL of suspension (WHO 1999).

The numbers of motile spermatozoa were counted under the microscope, and the result was expressed as percentage after counting 100 spermatozoa in each field as per the method of World Health Organization (WHO 1999). The count of viable spermatozoa in epididymal suspension sample was performed by the eosin-nigrosin staining according to the standard protocol (WHO 1999).

### Assessment of testicular androgenic key enzyme activities

Activities of testicular androgenic Δ5, 3β-HSD and 17β-HSD key enzyme were measured spectrophotometrically using the supernatant of testicular homogenate (Jarabak et al. 1962; Talalay 1962).

### Estimation of testicular cholesterol level

Spectrophotometric estimation of testicular cholesterol level was performed using the protocol of the kit supplied by Angstrom Biotech Pvt. Ltd., Makarpura Vadodara, Gujarat, India (Allain et al. 1974).

### Assay of serum testosterone level

Serum level of testosterone was quantified in ELISA reader following the immune enzymatic method (Srivastava 2000). Sample or standard of 10 μL of each was dispensed into micro well followed by mixing of 100 μL of enzyme conjugate containing horse-radish peroxidase (HRP). The strips were incubated for 60 min at 37 °C. The reaction solution was decanted forcefully from all the wells followed by three washings using washing buffer. Tetramethyl benzidine (TMB) and hydrogen peroxide (H_2_O_2_) substrate containing chromogen 100 μL each were added into the micro well. After the scheduled time, the reaction was terminated by addition of stop solution supplied in the kit. The absorbance of standards and samples were noted against the blank.

### Estimation of seminal plasma fructose level

The seminal plasma fructose level was quantified according to the standard method (Lu et al. 2007). The seminal plasma was deproteinised by adding 50 μL of zinc sulphate and 50 μL of sodium hydroxide to make a total dilution of seminal plasma 1:16, followed by centrifugation at 4000 *g* for 15 min. This clear supernatant of 200 μL was used for the analysis.

### Estimation of catalase, peroxidase activities

The activities of catalase and peroxidase in sperm pellet were assessed biochemically following the standard protocol (Chatterjee et al. 2009).

### Estimation of end products of lipid peroxidation (CD and TBARS)

For quantification of end products of lipid peroxidation, i.e., conjugated diene (CD) and thiobarbituric acid reactive substances (TBARS), the sperm pellet was homogenized at the concentration of 50 mg/mL in 0.1 M of ice-cold phosphate buffer (pH 7.4), and the homogenates were centrifuged at 10,000 *g* for 5 min at 4 °C. Supernatant was used for the estimation of the levels of CD and TBARS following the standard method (Ohkawa et al. 1979).

### Histological study

Testes were embedded in paraffin block, sectioned at 5 μm thickness and stained with hematoxylin-eosin. The stained slides were scanned under high power objective of computer attached trinocular microscope. A photograph of a particular field of the concerned section was snapped. Seminiferous tubular diameter (STD) was measured with the ‘De Winter Calipro-3.0’ Software. Quantitative analysis of spermatogenesis was carried out at the stage VII of the seminiferous epithelial cell (Leblond and Clermont 1952).

### Effective dose 50 (ED_50_) and lethal dose (LD_50_) study

Values of LD_50_ of individual extract and ‘Contracept-TM’ were measured from the plot of log doses verses probits followed by transformation of percentage mortalities to Probit units. Similarly values of ED_50_ of individual extracts and ‘Contracept-TM’ were measured by plotting the response percentage along the Y-axis and doses of lowest, intermediates and highest along the X-axis.

### Statistical analysis

Data were expressed as mean ± SEM. ‘Analysis of Variance (ANOVA)’ followed by ‘Multiple Comparison Student’s two tail *t*-test’ was used for statistical analysis of data (Sokal and Rohle 1997) and *p* < 0.05 was considered as the level of significance.

## Results

### Body weights and organo-somatic indices

The oral administration of the extract or CPA did not cause any significant change in the body weight in comparison to the control (Table 1). Testiculo-somatic, seminal vesiculo-somatic and epididymo-somatic indices were significantly decreased in all the treated groups (44%; 60%; 60% in ‘Contracept-TM’, 28%; 33%; 34% in *T. chebula* extract, 26%; 39%; 31% in *M. balbisiana* extract and 36%; 75%; 63% in CPA treated) in respect to the control group. The levels of these parameters were significantly decreased in ‘Contracept-TM’ and CPA-treated groups in respect to *T. chebula* or *M. balbisiana*-treated group (Table 1). However, no significant difference in the body weight as well as the levels of said organo-somatic indices except the epididymo-somatic index was noted between the CPA and ‘Contracept-TM’ treated groups (Table 1).

**Table 1.** Effect of separate extract or ‘Contracept-TM’ of *T. cheb*ula, *M. balbisi*ana or CPA on body weight, testiculo-somatic, epididymal somatic and seminal vesiculo-somatic indices in male rat.

Groups	Body weight (g)		Relative organ weights (g/100 g body weight)		
	Initial	Final	Testis	Epididymis	Seminal vesicle
Control	125.34 ± 3.54^a^	138.42 ± 3.65^a^	1.64 ± 0.07^a^	0.48 ± 0.04^a^	0.41 ± 0.02^a^
*T. chebula* treated	132.42 ± 3.25^a^	144.32 ± 2.41^a^	1.18 ± 0.05^b^	0.32 ± 0.03^b^	0.27 ± 0.04^b^
*M. balbisiana* treated	128.64 ± 2.64^a^	142.62 ± 2.54^a^	1.21 ± 0.06^b^	0.29 ± 0.05^b^	0.28 ± 0.03^b^
Contracept-TM	130.72 ± 2.74^a^	145.85 ± 3.34^a^	0.91 ± 0.07^c^	0.19 ± 0.04^c^	0.16 ± 0.04^c^
CPA treated	126.48 ± 3.21^a^	137.46 ± 2.65^a^	1.04 ± 0.06^c^	0.12 ± 0.03^d^	0.15 ± 0.03^c^

Data represented as Mean ± SEM, *n* = 6. ‘ANOVA followed by Multiple Comparison Student’s two tail *t*-test’. Data with different superscripts (a, b, c, d) differ from each other significantly, *p* < 0.05.

### Epididymal sperm count, motility and viability

Number of epididymal sperm along with sperm motility and viability were decreased significantly in ‘Contracept-TM’ (57.41%; 38.09%; 34.84%) or *T. chebula* (30.67%; 23.80%; 22.69%) or *M. balbisiana* (34.56%; 25.51%; 25.54%) extract treated or CPA (64.55%; 41.51%; 37.09%) treated group in respect to the vehicle control. ‘Contracept-TM’ or CPA treated group showed a significant level of diminution in these parameters compared to *T. chebula* or *M. balbisiana*-treated group (Table 2). Moreover, insignificant diminution in the values of these parameters was noted when comparison was made between ‘Contracept-TM’ and CPA-treated groups (Table 2).

**Table 2.** Cauda epididymal sperm count, motility, viability, levels of serum testosterone, testicular cholesterol and seminal vesicular fructose in separate extract or ‘Contracept-TM’ of *T. cheb*ula, *M. balbisi*ana or CPA-treated group.

Group	Sperm count(million /ml)	Sperm motility (%)	Sperm viability (%)	Serum testosterone (ng/ml)	Testicular cholesterol(mg/g of tissue)	Seminal vesicularfructose (µg/g of tissue)
Control	26.18 ± 1.68^a^	84.28 ± 1.29^a^	86.29 ± 1.72^a^	14.28 ± 0.5^a^	30.28 ± 1.15^a^	0.082 ± 0.004^a^
*T. chebula* treated	18.15 ± 1.75^b^ (30.67% ↓)	64.22 ± 1.68^b^ (23.80% ↓)	66.71 ± 1.62^b^ (22.69% ↓)	10.26 ± 0.8^b^ (28.15% ↓)	36.22 ± 1.42^b^ (19.61% ↑)	0.058 ± 0.003^b^ (29.26% ↓)
*M. balbisiana* treated	17.13 ± 1.52^b^ (34.56% ↓)	62.78 ± 1.52^b^ (25.51% ↓)	64.25 ± 1.48^b^ (25.54% ↓)	9.18 ± 0.6^b^ (35.71% ↓)	38.26 ± 1.06^b^ (26.35% ↑)	0.057 ± 0.003^b^ (30.48% ↓)
Contracept-TM	11.15 ± 1.61^c^ (57.41% ↓)	52.17 ± 1.98^c^ (38.09% ↓)	56.22 ± 1.79^c^ (34.84% ↓)	6.25 ± 0.5^c^ (56.23% ↓)	44.58 ± 1.05^c^ (47,22% ↑)	0.031 ± 0.004^c^ (62.19% ↓)
CPA treated	9.28 ± 1.78^c^ (64.55% ↓)	49.29 ± 1.76^c^ (41.51% ↓)	54.28 ± 1.82^c^ (37.09% ↓)	5.22 ± 0.6^c^ (63.44% ↓)	45.22 ± 1.37^c^ (49.33% ↑)	0.036 ± 0.004^c^ (56.09% ↓)

Data expressed as Mean ± SEM, *n* = 6. ‘ANOVA followed by Multiple Comparison Student’s two tail *t*-test’. Percentage has been calculated in respect to control. Data having different superscripts (a, b, c) differ from each other significantly, *p* < 0.05.

### Testicular Δ^5^, 3β-HSD and 17β-HSD

**Figure 1.**
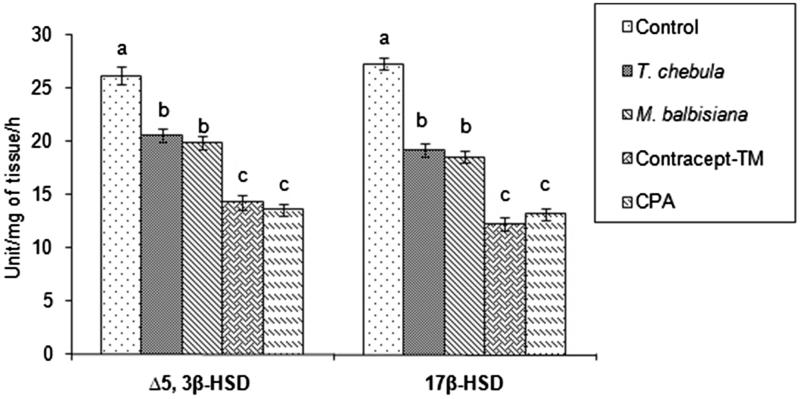
Activities of testicular Δ5, 3β-HSD and 17β-HSD after treatment with separate extract or ‘Contracept-TM’ of *T. chebula*, *M. balbisiana* or CPA. Bars expressed in terms of Mean ± SEM, *n* = 6. ‘ANOVA followed by Multiple Comparison Student’s two tail *t*-test’. Bars with different superscripts (a, b, c) differ from each other significantly, *p* < 0.05.

Testicular Δ^5^, 3β-HSD and 17β-HSD activities were decreased significantly in all the extracts (45.44%; 55.08% in ‘Contracept-TM’, 21.25%; 29.75% in *T. chebula* extract treated, 24.11%; 32.16% in *M. balbisiana* extract treated) treated groups or CPA treated (48%; 51.68%) group in comparison with the vehicle control group (Figure 1). Significant inhibition in these two steroidogenic key enzyme activities was noted in the ‘Contracept-TM’ or CPA-treated group in respect to the *T. chebula* or *M. balbisiana*-treated group (Figure 1).

### Testicular cholesterol

The level of the testicular cholesterol was elevated significantly in *T. chebula* (19.61%) or *M. balbisiana* (26.35%) or ‘Contracept-TM’ (47.22%) or CPA-treated (49.33%) group in respect to the control group (Table 2). The level of this parameter was increased significantly in ‘Contracept-TM’ treated group in comparison with *T. chebula* or *M. balbisiana* treated group. No significant difference was noted in the level of testicular cholesterol when comparison was made between the ‘Contracept-TM’ and CPA-treated groups (Table 2).

### Testosterone

Serum testosterone level was decreased significantly in all the extracts (56.23% in ‘Contracept-TM’ treated, 28.15% in *T. chebula* treated and 35.71% in *M. balbisiana* treated) or CPA treated (63.44%) group as compared with the vehicle control group (Table 2). After ‘Contracept-TM’ treatment, serum level of testosterone was significantly lowered in comparison to the *T. chebula* or *M. balbisiana* treated group. The level of serum testosterone did not differ significantly when comparison was made between ‘Contracept-TM’ and CPA-treated groups (Table 2).

### Seminal vesicular fructose

A significant diminution in the level of seminal vascular fructose was noted in CPA (56.09%) and all the extract (62.19% in ‘Contracept-TM’ treated, 29.26% in *T. chebula* treated, 30.48% in *M. balbisiana* treated) treated groups in comparison to the vehicle control. No significant difference in the level of this parameter was noted in comparison between the *T. chebula* and *M. balbisiana*-treated groups (Table 2). However, the level of this parameter was significantly decreased in the ‘Contracept-TM’ treated group in comparison to the *T. chebula* or *M. balbisiana* treated group. No significant change in the level of this parameter was noted between CPA and ‘Contracept-TM’ treated groups (Table 2).

### Oxidative stress in sperm pellet

**Figure 2.**
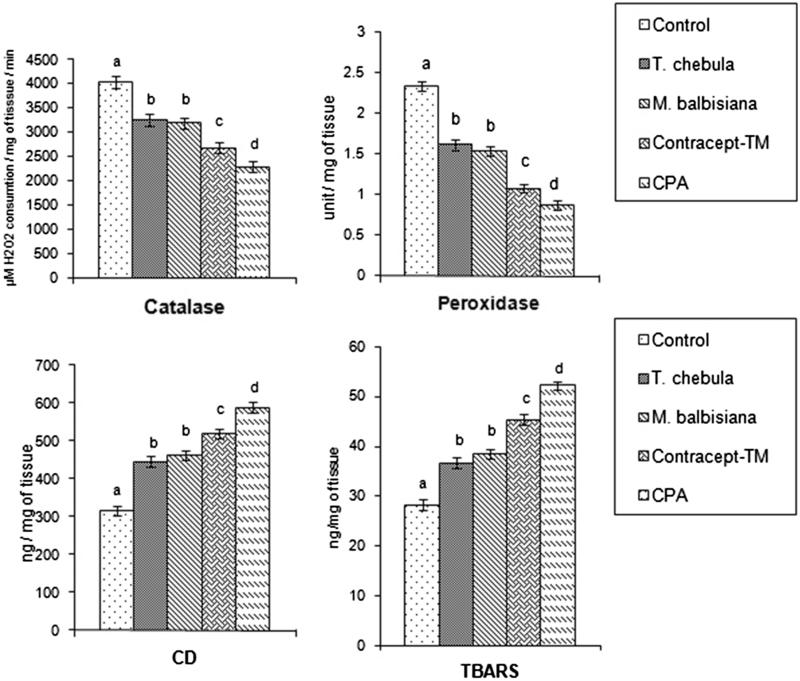
Catalase, peroxidase activities and levels of CD, TBARS in sperm pellet in *T. chebula*, *M. balbisiana* separate extract or ‘Contracept-TM’ or CPA-treated group. Bars expressed in terms of Mean ± SEM, *n* = 6. ‘ANOVA followed by Multiple Comparison Student’s two tail *t*-test’. Bars with different superscripts, i.e., (a, b, c, d) differ from each other significantly, *p* < 0.05.

Catalase and peroxidase activities in sperm pellet were decreased significantly in all the extract (33.55%; 53.84% in ‘Contracept-TM’, 19.06; 30.76% in *T. chebula* and 20.82; 34.18% in *M. balbisiana* treated), and CPA-treated (43.29%; 62.82%) groups in respect to the vehicle control group (Figure 2). However, ‘Contracept-TM’ treatment produced a significant inhibition in the activities of both the enzymes in comparison to the individual extract treated group. The significant diminution was also noted in the levels of these parameters in CPA-treated group when compared with ‘Contracept-TM’ treated group (Figure 2).

Thiobarbituric acid reactive substances (TBARS) and conjugated diene (CD) are the main end products of lipid peroxidation. After treatment with *T. chebula* or *M. balbisiana* or ‘Contracept-TM’ or CPA, levels of TBARS and CD in sperm pellet were elevated significantly in all the extract (41.25%; 29.69% in *T. chebula*, 46.62%: 35.82% in *M. balbisiana* treated, 64.38%; 60.41% in ‘Contracept-TM’ treated) and CPA treated (86.73%; 84.99%) groups in comparison to the vehicle control group. Insignificant changes in the levels of these parameters were noted between *T. chebula,* and *M. balbisiana* treated groups (Figure 2). However, the levels of these two parameters were elevated significantly in ‘Contracept-TM’ treated group in respect to *T. chebula* or *M. balbisiana* treated group. Levels of TABRS and CD in sperm pellet were significantly increased in CPA treated group in respect to ‘Contracept-TM’ treated group (Figure 2).

### Germ cells at stage VII of seminiferous epithelial cycle

**Figure 3.**
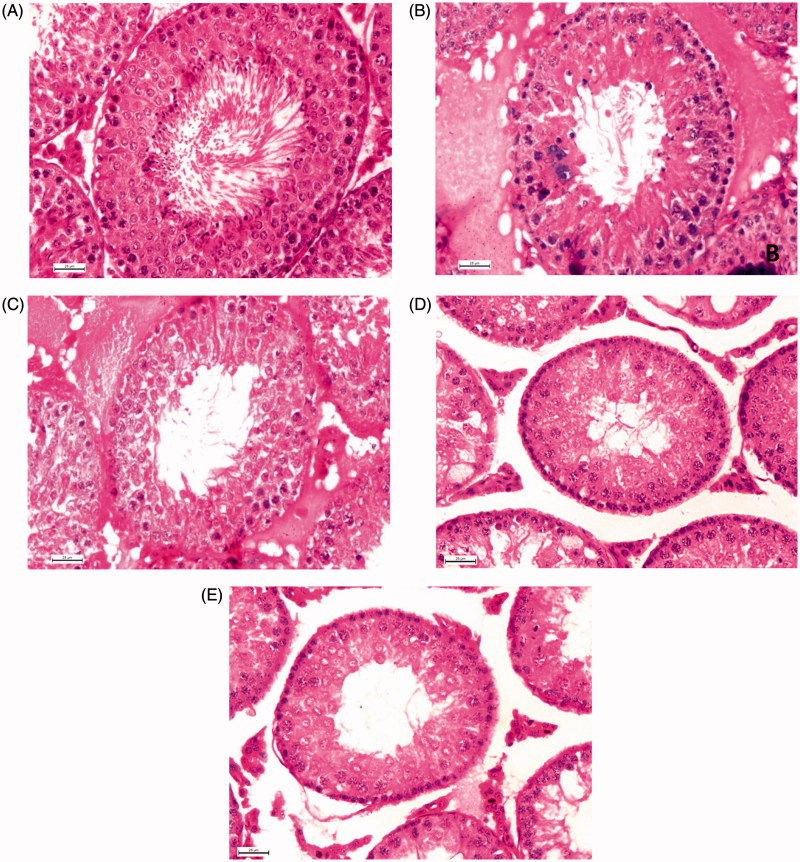
Histology of testis 400 X (Hematoxylin Eosin Stain). Representative microphotographs of a vehicle control (A), *T. chebula* (B), *M. balbisiana* (C), ‘Contracept-TM’ (D) and CPA (E) treated rat. Vehicle control group rat showed normal spermatogenesis at stage VII and STD. Different individual extract, ‘Contracept-TM’ and CPA-treated groups showed significant decrease in the number of different generations of germ cells at stage VII and STD in respect to the vehicle control group (*p* < 0.05).

Quantitative study of hormone sensitive germ cells at stage VII of seminiferous epithelial cell cycle revealed a significant diminution in the numbers of ASg, pLSc, mPSc and 7SD after treatment with CPA (49.09%; 55.30%; 47.28%; 51.59%) or individual extract (36.36%; 19.11%; 28.65%; 29.65% in *T. chebula* and 30.30%; 25.48%; 22.63%; 24.41% in *M. balbisiana*) or ‘Contracept-TM’ (44.24%; 49.69%; 42.46%; 46.12%) in comparison to the vehicle control group (Table 3). The numbers of above-mentioned germ cells were diminished significantly when the animals were treated with ‘Contracept-TM’ in comparison to the individual extract treated groups (Table 3). No significant difference in the count of above-mentioned germ cells was noted between the ‘Contracept-TM’ and CPA-treated groups (Table 3 and Figure 3).

**Table 3.** Effect of *T. cheb*ula, *M. balbisi*ana in separate extract or ‘Contracept-TM’ or CPA on the number of different generations of germ cells at stage VII of seminiferous epithelial cell cycle.

Groups	ASg	pLSc	mPSc	7Sd	STD ×400(µm)
Control	1.65 ± 0.06^a^	19.62 ± 0.59^a^	18.25 ± 0.88^a^	64.50 ± 1.79^a^	554.35 ± 12.08^a^
*T. chebula* treated	1.05 ± 0.07^b^ (36.36% ↓)	15.87 ± 0.71^b^ (19.11% ↓)	13.02 ± 0.61^b^ (28.65% ↓)	45.37 ± 1.82^b^ (29.65% ↓)	423.19 ± 18.23^b^ (23.60% ↓)
*M. balbisiana* treated	1.15 ± 0.06^b^ (30.30% ↓)	14.62 ± 0.88^b^ (25.48% ↓)	14.12 ± 0.78^b^ (22.63% ↓)	48.75 ± 1.58^b^ (24.41% ↓)	438.37 ± 12.60^b^ (20.92% ↓)
Contracept-TM	0.92 ± 0.08^c^ (44.24% ↓)	9.87 ± 0.93^c^ (49.69% ↓)	10.50 ± 0.82^c^ (42.46% ↓)	34.75 ± 1.49^c^ (46.12% ↓)	328.14 ± 13.53^c^ (40.83% ↓)
CPA treated	0.84 ± 0.06^c^ (49.09% ↓)	8.77 ± 0.78^c^ (55.30% ↓)	9.62 ± 0.68^c^ (47.28% ↓)	31.22 ± 1.62^c^ (51.59% ↓)	342.22 ± 15.64^c^ (38.30% ↓)

Data expressed as Mean ± SEM, *n* = 6. ‘ANOVA followed by Multiple Comparison Student’s two tail *t*-test’. Percentage has been calculated in respect to the vehicle control. Data having different superscripts (a, b, c) differ from each other significantly, *p* < 0.05.

### Testicular histometry

Histometric studies indicated a significant decrease in the STD in separate as well as ‘Contracept-TM’ (40.80% in ‘Contracept-TM’ treated, 23.66% in *T. chebula* treated; 20.92% in *M. balbisiana* treated) or CPA treated (38.26%) group in comparison to the vehicle control group (Table 3). Significant diminution was also noted in CPA treated or ‘Contracept-TM’ treated group when comparison was made with individual extract treated group. No significant difference in the level of this parameter was noted between the *T. chebula* and *M. balbisiana* treated groups as well as ‘Contracept-TM’ treated and CPA-treated groups (Table 3 and Figure 3).

### LD_50_ and ED_50_ values of extracts

By calculating probit values and their transformations to percentage mortalities, it has been noted that LD_50_ values of *T. chabula* was 5.8 g, *M. balbisiana* was 6.3 g and of ‘Contracept-TM’ was 6 g/100 g body weight. ED_50_ values were computed as *T. chebula* extract – 40 mg, *M. bulbisiana* – 48 mg and ‘Contracept-TM’ – 40 mg/100 g body weight.

## Discussion

Comparative study of accessory sex organo-somatic indices indicated that the ‘Contracept-TM’ is most effective out of the two separate extract used here and is similar with CPA. The significant diminution in the relative weights of testiculo-somatic, seminal vesiculo-somatic and epididymo-somatic indices in ‘Contracept-TM’ treated group in respect to the individual extract treated groups may be due to inhibition in testosterone biosynthesis by herb–herb interaction (Patil et al. 1998). Testicular inhibitory function have been further supported here by the diminution in epididymal sperm count and by the low number of different generations of germ cells of spermatogenic cells cycle at stage VII as both are the reflectors of spermatogenesis (Paul et al. 2009). The significant decrease in the values of both sensors after treatment with ‘Contracept-TM’ in respect to other extract treated groups once again supported that ‘Contracept-TM’ is most effective for imposition of antitesticular activity than the separate extract. The inhibition in spermatogenesis by extract, and CPA may be due to low-testicular androgenesis as testosterone is one of the important regulators of sperm production (McLachlan et al. 2002) which has been further supported here from the histometric study like STD, as STD is one of the indicators of serum testosterone (Ghosh et al. 2002). Testicular Δ5, 3β-HSD and 17β-HSD are the key enzymes of testicular steroidogenesis (Murono and Payne 1979) and the activities of these key enzymes are under the control of pituitary gonodotropins (Hall and Eik-nes 1963). The results of these steroidogenic key enzymes indicated that the treatment with ‘Contracept-TM’ was the most effective than individual extract as comparable with CPA, a standard antitesticular drug. Inhibition in testicular androgenic key enzymes suggested that the extract inhibits the testicular activities by modulating the pituitary testicular axis. The significant decrease in testicular androgenesis by treatment with ‘Contracept-TM’ or CPA was further supported here from the estimation of testicular cholesterol and serum testosterone levels. The cholesterol is the mother molecule of steroids, one of the reflectors of androgenesis (Lin et al. 1995) and testosterone is the final product of androgenesis (Morris and Chaikoef 1959). After treatment with ‘Contracept-TM’, significant elevation in testicular cholesterol level along with diminution in serum testosterone level in comparison to other extract again supported that the effective treatment was ‘Contracept-TM’ identical with CPA-treated group. Seminal vesicular fructose level is the important biosensor of testosterone (Ghosh et al. 1990). Significant diminution in the level of this parameter was noted when the animals were subjected to treatment of ‘Contracept-TM’ in comparison to separate extract which strengthen the polyherbal concept of contraceptive development.

To find out whether the extract inhibits the testicular activity by inducing oxidative stress as an alternative way, several oxidative stress parameters were considered beside pituitary testicular axis assessment. Activities of catalase, peroxidase and quantification of the CD and TABRS levels in sperm pellet, which are the indicators of oxidative stress, were changed significantly in favour of oxidative stress generation. This result supports the fact that the extract may additionally affect male reproduction by developing oxidative stress (Saleh and Agarwal 2002) and it may have some direct spermicidal effect on the germ cells. Oxidative stress additionally affects the sperm motility and sperm viability (Alvarez and Storey 1995). Above hypo-testicular activities by the phytomolecule present in above plants may be due to the presence of ferulic acid, C_16_, C_18_ fatty acid and polyphenols in *M. bulbisiana* seeds (Graven et al. 1996) and gallic acid, ellagic acid, chebulinic acid, ethyl gallate, punicalagin, luteolin and tannic acid present in *T. chebula* fruit (Lee et al. 2006). From the literature review it has been mentioned that CPA may impose oxidative stress in the same way (Siddique and Afzal 2005). About the mechanism of action of the phytomolecules present in the polyherbal contraceptive i.e., ‘Contracept-TM’, two hypothesis may be put forwarded. The phytomolecules may directly inhibit the testicular androgenesis and thereby oligospermia related infertility may be developed. Other possibility is that the phytomolecules may exert oxidative stress on testicular tissue including germ cells and thereby spermiological sensor levels are lowered which ultimately results male infertility. Hope the actual mechanism may be unfolded from the upcoming investigation in short time. Regarding the efficacy of polyherbal formulation in this domain the hypothesis may be developed that phytomolecule(s) which is present in one extract may trigger the activity of the other phytomolecule(s) present in the extract of other plant. This synergistic effect for contraceptive efficacy is in parallel with our previous observation where polyherbal formulation is more effective than individual via herb–herb interaction (Chatterjee et al. 2009).

## Conclusions

The results of the present investigation highlighting that the composite extract of *T. chebula* and *M. balbisiana* named as ‘Contracept-TM’ has most promising hypo-testicular activity compared to the standard antitesticular drug like CPA.
